# Impact of Acute Respiratory Infections on Medical Absenteeism Among Military Personnel: Retrospective Cohort Study

**DOI:** 10.2196/69113

**Published:** 2025-04-18

**Authors:** Premikha M, Jit Khong Goh, Jing Qiang Ng, Adeliza Mutalib, Huai Yang Lim

**Affiliations:** 1 Force Health Group, HQ Medical Corps, Singapore Armed Forces, 701 Transit Road, #02-03Singapore, 778910, Singapore; 2 Saw Swee Hock School of Public Health, National University of Singapore, Singapore, Singapore

**Keywords:** respiratory infections, military, epidemiology, public health, surveillance

## Abstract

**Background:**

Acute respiratory infections (ARI) are a significant challenge in military settings due to close communal living, which facilitates the rapid transmission of pathogens. A variety of respiratory pathogens contribute to ARI, each varying in prevalence, severity, and impact on organizational productivity. Understanding and mitigating the impact of ARI is critical for optimizing the health of military personnel and maintaining organizational productivity.

**Objective:**

This retrospective study of surveillance data aims to identify pathogens causing ARI among servicemen and determine which pathogens contribute most to medical absenteeism, defined as the combined duration of the issued medical certificate and light duty.

**Methods:**

From September 2023 to August 2024, anonymous nasopharyngeal swabs (BioFire FilmArray Respiratory Panel) were collected from Singapore Armed Forces servicemen presenting with ARI symptoms after a doctor’s consultation at a local military camp’s medical centre. The presence of fever and duration of medical certificate and light duty were self-reported by Singapore Armed Forces servicemen.

**Results:**

A total of 1095 nasopharyngeal swabs were collected, of which 608 (55.5%) tested positive. The most common respiratory pathogen was human rhinovirus/enterovirus (HRV/HEV) in 303 (27.7%) individuals. The highest proportions of fever were observed in servicemen with influenza (62.8%, 27/43), SARS-CoV-2 (34.3%, 12/35), and parainfluenza (31.6%, 12/38). The odds of patients with influenza that have fever was 5.8 times higher than those of patients infected with HRV/HEV (95% CI 2.95‐11.40, *P*<.001). The median duration of medical certificate, light duty, and medical absenteeism were 0 (IQR 0), 2 (IQR 2) and 2 (IQR 0) days, respectively. The odds of patients with influenza having a medical certificate with duration ≥1 day was 5.34 times higher than those in patients with HRV/HEV (95% CI 2.63‐10.88, *P*<.001). No significant differences in the duration of medical absenteeism were found between HRV/HEV and other pathogens.

**Conclusions:**

Compared to HRV/HEV, influenza infections were significantly associated with longer medical certificate duration. Nonetheless, there were no significant differences in the overall duration of medical absenteeism across pathogens, as servicemen infected with other pathogens were given light duty instead. These findings emphasize the need for pathogen-agnostic ARI measures. While influenza vaccinations are already mandatory for servicemen in local military camps, encouraging additional public health measures (eg, mask-wearing among symptomatic servicemen, COVID-19 vaccinations, therapeutics) can further reduce ARI incidence, minimize the duration of medical absenteeism, and mitigate the impact on organizational productivity.

## Introduction

### Background

Acute respiratory infections (ARI) pose a significant challenge in military settings like the Singapore Armed Forces (SAF), where close quarters living and shared communal spaces, equipment, and furniture could facilitate the rapid transmission of respiratory pathogens [1,2]. Singapore’s high humidity and tropical climate further exacerbate these risks, promoting the survival and spread of respiratory viruses [3]. These conditions not only increase ARI risk and impact the health of military personnel but also reduce organizational productivity due to medical absenteeism [1]. Despite ongoing surveillance, gaps remain in understanding how specific ARI pathogens impact medical absenteeism and readiness in high-density military environments.

A comprehensive understanding of the full spectrum of ARI pathogens is critical. While influenza is known for causing significant morbidity and mortality, HRV is typically the most prevalent pathogen and can lead to chronic infections and secondary bacterial infections, particularly in immunocompromised individuals [4]. A population-wide study in the United States found that the hospitalization burden was higher for HRV/HEV compared to influenza among adults aged <65 years [5,6]. Additionally, bacterial pathogens such as *Streptococcus pneumoniae* and *Mycoplasma pneumoniae* can act as primary agents or secondary infections [7,8]. A 2016 study in Singapore military recruits further emphasized the diversity of ARI pathogens, with 41% of 2647 recruits presenting with febrile respiratory illness (FRI) were predominantly caused by adenovirus and influenza viruses [9]. Among the remaining nonfebrile ARI individuals, rhinovirus accounted for 47% [9]. These findings highlight the need for expanded surveillance to capture the full spectrum of pathogens, beyond influenza to assess their contribution to the ARI burden in military settings.

Furthermore, it is unclear whether current influenza vaccination efforts sufficiently address the impact of influenza in military settings. Historical and contemporary data underscore the disproportionate impact of influenza on military populations, with attack rates reaching as high as 37‐45% during outbreaks [10]. During the 1918 influenza pandemic, military personnel living in confined spaces faced significantly higher morbidity and mortality rates compared to civilian populations [10]. Recent outbreaks further highlight the heightened vulnerability of military personnel; a 2013 influenza A (H1N1) outbreak in a Swiss military camp infected 31% of soldiers, while a 2009 influenza A outbreak in a Peruvian Navy ship affected 22% of crew members [11,12]. Recognizing influenza as a significant cause of ARI with substantial morbidity, the SAF has conducted annual Northern Hemisphere influenza vaccination drives since 2015, achieving over 97% coverage in 2023 [2]. Additionally, the SAF conducts Southern Hemisphere influenza vaccinations for high-risk servicemen (eg, health care workers, medically vulnerable individuals), and strongly encourages them to receive additional doses of the COVID-19 vaccines, aligning with national guidelines [13]. Despite these measures, there is limited understanding of whether SAF’s influenza vaccination programme sufficiently mitigate influenza’s prevalence and severity in the SAF. These efforts are also insufficient to address the broader ARI burden, as evidenced by the prevalence of other ARI pathogens, highlighting the need for pathogen-specific data to understand the impact of various ARI pathogens on medical absenteeism.

Another critical gap is the limited understanding of the differential impact of febrile and nonfebrile ARI on medical absenteeism in military settings. The FRI, characterized by the presence of fever (≥38°C), serves as a marker of severity and reflects a robust immune response to more virulent pathogens [14]. Influenza and SARS-CoV-2 often cause FRI, whereas HRVs typically cause nonfebrile illnesses [3,15,16]. This distinction is crucial not only for clinical management but also for informing public health strategies aimed at controlling the spread of more severe FRI. However, findings from the Singapore military recruit study in 2016 showed that ARI cases without fever constituted the majority (59%) of respiratory illnesses and contributed significantly to the overall burden, emphasizing the need to account for both FRI and nonfebrile ARI in public health strategies [9]. Clinical management for ARI is mostly supportive, making vaccines critical for certain pathogens such as influenza and SARS-CoV-2. Recent events such as the 2023 ARI outbreak in the Philippines military that affected 20% of military students due to low influenza vaccine coverage [17], highlight the importance of maintaining high vaccine uptake. However, they also underscore the need to explore additional public health measures to reduce the broader ARI burden in military settings.

### Study Objectives

Therefore, this study aimed to address these gaps by reviewing the results of the respiratory pathogen surveillance study from a local military camp to answer the following questions:

1. What is the distribution of pathogens responsible for ARI among SAF servicemen?
2. Which pathogens had the greatest impact on duration of medical absenteeism and how can this inform public health interventions to mitigate the impact?

By addressing these questions, this study seeks to determine if additional public health measures need to be instituted at local camps, beyond the existing influenza vaccinations. Understanding and mitigating the impact of ARI is critical for reducing medical absenteeism and optimizing organizational productivity.

## Methods

### Study Design and Recruitment

This retrospective study of surveillance data analyzed ARI cases from September 2023 to August 2024 at the medical centre of a local military camp in Singapore, the primary health care facility for servicemen. The camp’s population primarily consists of young adult males, both conscripted and regular military personnel, who live in high-density accommodations conducive to ARI transmission. The medical centre typically manages 100-150 servicemen per day with acute conditions. The study used a convenience sampling approach, with recruitment conducted solely at the medical centre. Doctors screened servicemen presenting with ARI symptoms (eg, cough, sore throat, rhinorrhea) during routine consultations. Eligible servicemen were provided a standardized explanation of the study and invited to participate voluntarily. Those who agreed to participate were directed to the study team for data collection. Inclusion criteria included servicemen who (1) presented with at least one ARI symptom and (2) be willing to provide nasopharyngeal swabs for diagnostic testing. Exclusion criteria included servicemen diagnosed with other acute concurrent illnesses or those unwilling to provide swabs.

Anonymized nasopharyngeal swabs were collected from all eligible participants by the study team and tested using the BioFire FilmArray Respiratory Panel (BioFire Diagnostics), a multiplex polymerase chain reaction assay capable of detecting 22 respiratory pathogens. These pathogens were grouped into: (1) coronaviruses (coronavirus 229E, HKU1, NL63, OC43), (2) SARS-CoV-2, (3) human metapneumovirus, (4) HRV/HEV, (5) influenza (influenza A, A/H1, A/H3, A/H1-2009, B), (6) parainfluenza (parainfluenza 1, 2, 3, 4), (7) respiratory syncytial virus, (8) adenovirus, and (9) bacteria (ie, *Bordetella parapetussis*, *Bordetella pertussis*, *Chlamydia pneumoniae*, *Mycoplasma pneumoniae*) for analysis. Pathogen testing was conducted immediately after swab collection using our mobile BioFire systems, which were serviced annually to minimize testing errors. The presence of fever (≥38°C) and duration of medical certificate and light duty were recorded through self-reported surveys administered directly by the surveillance team to ensure consistency in data collection. Our outcome of interest was duration of medical absenteeism, defined as the combined duration of medical certificate and light duty, representing the total period during which servicemen were unable to perform their primary jobs at full capacity. Servicemen with medical certificates were fully exempted from their duties, while those on light duty were partially exempted.

### Statistical Analysis

Descriptive statistics were presented as mean (SD) for continuous variables and distribution (n, %) for categorical variables. Invalid swabs, coinfections, and bacterial cases were excluded from the analysis. Coinfections were defined as the presence of more than one pathogen detected in a single swab. Excluding coinfections aimed to minimize potential bias and ensured clearer attribution of outcomes (eg, fever, duration of medical absenteeism) to individual pathogens. Bacterial cases, which included those positive for bacterial pathogens such as *Mycoplasma pneumoniae*, were excluded due to the small number of cases detected during the study period.

Comparative analyses were performed to examine the differences in the prevalence of fever, duration of medical certificate, light duty, and duration of medical absenteeism among different pathogens using Pearson’s *χ*^*2*^ test of independence. Logistic regression was then performed for the outcomes of interest: (1) medical certificate (medical certificate ≥1 day), (2) light duty (>1 day), and (3) medical absenteeism (>1 day) to quantify outcome differences across pathogens. Human rhinovirus/human enterovirus was selected as the reference group due to its high prevalence in the study population, contributing to the stability of regression estimates. While “negative” cases could serve as an alternative reference group, they introduce heterogeneity, as “negative” indicates the absence of detectable pathogens by the BioFire Respiratory Panel, rather than the absence of infection. The Hosmer-Lemeshow goodness-of-fit test was used to ensure that all models characterized the data distribution accurately. Data were analyzed using Stata (version 17.0, StatCorp LLC) at a 5% significance level.

### Ethical Considerations

This study was granted exemption from ethical review by the institutional review board (IRB) of the Defence Science Organisation–SAF (DSO-SAF IRB; reference no.: 0006/2024), as it was retrospective in nature, with only nonidentifiable parameters extracted and all data anonymized prior to recording and analysis. The IRB exemption allowed for secondary analysis without additional consent. All data were deidentified to ensure privacy and confidentiality, and no personally identifiable information or images of participants were included in this manuscript. No compensation was provided to the study participants.

## Results

### ARI Pathogen Distribution

From September 2023 to August 2024, a total of 1095 servicemen with ARI symptoms were included in the study. The median age of participants was 20 (IQR 2.0, range 17-31) years, with 89.9% of participants aged 18‐21 years, reflecting a relatively homogenous age distribution. Additionally, 99.8% of participants were male. Of the 1095 nasopharyngeal swabs collected, 608 (55.5%) tested positive for at least one respiratory pathogen. The distribution of pathogens is shown in (Table 1). The most common pathogen was HRV/HEV (n=303, 27.7%). There were 69 (6.3%) coinfections; specifically, HRV/HEV co-occurred with other pathogens in 50 cases (72.4%), influenza co-occurred in 6 cases (8.7%), and both HRV/HEV and influenza co-occurred in 7 cases (10.1%). Coinfections were excluded from further analysis to minimize bias and ensure a clearer attribution of the study outcomes to specific pathogens. Additionally*, Mycoplasma pneumoniae* (n=3) and adenovirus (n=1) were excluded, along with 31 (2.83%) invalid swabs.

**Table 1.** Nasopharyngeal swab test results among servicemen with acute respiratory infections in the Singapore Armed Forces from September 2023 to August 2024.

Nasopharyngeal swab test results	Number of swabs (N=1095), n (%)^a^
Positive	608 (55.5)^b^
Coronavirus^c^	68 (3.2)
SARS-CoV-2	35 (5.8)
Human metapneumovirus	37 (3.4)
Human rhinovirus/enterovirus	303 (27.7)
Influenza	43 (3.9)
Parainfluenza	38 (3.5)
Respiratory synctial virus	11 (1.0)
Adenovirus	1 (0.1)
Bacteria^d^	3 (0.3)
Coinfections	69 (6.3)
Negative	456 (41.6)
Invalid	31 (2.8)

a The percentages do not add up to 100% due to rounding.

b The percentages of all pathogens in the Positive test results category add up to 55.5%.

c Coronavirus includes seasonal coronaviruses (229E, HKU1, NL63, OC43) but does not include SARS-CoV-2, which is analyzed and reported as a separate pathogen group.

d *Bordetella parapetussis*, *Bordetella pertussis*, *Chlamydia pneumoniae*, *Mycoplasma pneumoniae.*

### Comparing the Prevalence of Fever (Severity) Across Respiratory Pathogen Groups

Among the 991 servicemen included for further analysis, fever was observed among 212 (21.4%). The highest proportions of fever were observed in servicemen with influenza (27/43, 62.8%), SARS-CoV-2 (12/35, 34.3%) and parainfluenza (12/38, 31.6%) (Table 2). There were significant differences in the proportions of servicemen with fever across the various pathogens (*χ*²_7_=65.9, *P*<.001). The odds of patients with influenza having fever were 5.8 times higher than those in patients infected with HRV/HEV (95% CI 2.95‐11.40; *P*<.001). There were no statistically significant associations between the other pathogen types and fever.

**Table 2.** Prevalence of fever among servicemen with specific respiratory pathogen groups from a retrospective study on acute respiratory infections among servicemen from the Singapore Armed Forces from September 2023 to August 2024.

Respiratory pathogen groups	Febrile cases/total infected (N=991), n (%)
Influenza	27/43 (62.8)
SARS-CoV-2	12/35 (34.3)
Parainfluenza	12/38 (31.6)
Human metapneumovirus	11/37 (29.7)
Human rhinovirus/enterovirus	70/303 (23.1)
Coronavirus^a^	13/68 (19.1)
Negative	66/456 (14.5)
Respiratory synctial virus	1/11 (9.1)

a Coronavirus includes seasonal coronaviruses (229E, HKU1, NL63, OC43) but does not include SARS-CoV-2, which is analyzed and reported as a separate pathogen group.

### Impact of ARI on Organizational Productivity

The median durations of medical certificates, light duty and medical absenteeism were 0 (IQR 0), 2 (IQR 2) and 2 (IQR 0) days, respectively.

- Medical certificate: There were significant differences in the proportions of servicemen with medical certificates ≥1 day across the various pathogens (χ²_7_=29, *P*<.001). The odds of patients with influenza having medical certificates ≥1 day was 5.34 times that of HRV/HEV patients (95% CI 2.63‐10.88; *P*<.001).
- Light duty: There were no significant differences in the proportion of servicemen with light duty >1 day across the various pathogens (*χ*²_7_=13.3, *P*=.07). However, patients with influenza had significantly lower odds of having light duty >1 day compared to that of HRV/HEV patients (odds ratio [OR] 0.33, 95% CI 0.17‐0.64); *P*=.001).
- Medical absenteeism: No significant differences in medical absenteeism were found between HRV/HEV and other pathogens (Table 3).

**Table 3.** Results of logistic regression of medical certificate ≥1 day, light duty >1 day and medical absenteeism >1 day on pathogens (n=991).

Outcomes and pathogens	Odds ratio (95% CI)	*P* value
Medical certificate ≥1 day		
Human rhinovirus/enterovirus	Reference	–^a^
Coronavirus	1.57 (0.75‐3.31)	.23
SARS-CoV-2	1.69 (0.65‐4.38)	.28
Human metapneumovirus	1.28 (0.47‐3.51)	.63
Influenza	5.34 (2.63‐10.88)	*<.001*
Parainfluenza	1.24 (0.45‐3.40)	.68
Respiratory syncytial virus	1.82 (0.38‐8.78)	.46
Negative	1.10 (0.69‐1.74)	.69
Light duty >1 day		
Human rhinovirus/enterovirus	Reference	–^a^
Coronavirus	0.93 (0.54‐1.61)	.80
SARS-CoV-2	0.60 (0.30‐1.22)	.16
Human metapneumovirus	0.83 (0.41‐1.69)	.61
Influenza	0.33 (0.17‐0.64)	*.001*
Parainfluenza	0.78 (0.39‐1.56)	.48
Respiratory syncytial virus	0.89 (0.25‐3.10)	.85
Negative	0.91 (0.67‐1.24)	.56
Medical absenteeism >1 day		
Human rhinovirus/human enterovirus	Reference	–^a^
Coronavirus	1.31 (0.68‐2.56)	.41
SARS-CoV-2	0.68 (0.32‐1.46)	.32
Human metapneumovirus	0.97 (0.44‐2.15)	.94
Influenza	1.03 (0.48‐2.19)	.94
Parainfluenza	0.87 (0.40‐1.88)	.73
Respiratory syncytial virus	1.30 (0.30‐6.64)	.67
Negative	0.98 (0.70‐1.38)	.91

a Not applicable.

## Discussion

### Principal Findings

This study provides critical insights into the epidemiology of ARI in a military setting, specifically among SAF servicemen in Singapore. It addresses a key gap in understanding the contribution of different ARI pathogens to medical absenteeism and highlights actionable strategies to mitigate ARI-related disruptions in military contexts. Over the study period, HRV/HEV was the most frequently detected pathogen (27.7%), aligning with previous research findings. Influenza was significantly associated with higher odds of fever, longer medical certificate duration, but the overall duration of medical absenteeism was similar to that of HRV/HEV. Even after adjusting for fever, influenza remained significantly associated with longer medical certificate duration (OR 2.81, 95% CI 1.28‐6.13, *P*=.01) compared to HRV/HEV, suggesting that other ARI symptoms (eg, sore throat, rhinorrhea), contribute to its more severe clinical presentation. This distinction is important as it provides evidence that influenza leads to more severe illness overall, beyond fever, compared to other ARI pathogens and makes servicemen more likely to receive medical certificates than light duties. Conversely, servicemen infected with other ARI pathogens—perceived as less severe and not significantly associated with fever—are more likely assigned to light duties instead. This reflects clinical practice, where servicemen with more severe ARI are allowed to recuperate at home for a longer duration, while those with milder ARI are required to return to work earlier but allowed to perform only light duties. These findings reinforce the importance of continuing robust influenza vaccination programmes in the SAF.

Despite these findings, our study demonstrated no significant differences in duration of medical absenteeism across pathogens, reflecting that ARIs, regardless of pathogen type, contribute substantially to absenteeism and operational disruptions. This highlights the need for pathogen-agnostic strategies targeting ARI prevention and management, rather than focusing solely on influenza in the SAF. The importance of nonpharmaceutical intervention (NPI) is further underscored by historical examples, such as the 1996 influenza A (H3N2) outbreak aboard a US Navy ship [18]. Despite 95% of the crew being vaccinated against influenza, the antigenic distinctness of the circulating strain led to an infection rate of 42%, demonstrating the limitations of vaccination alone and the critical need for complementary measures such as isolation protocols, improved ventilation, and rapid response strategies to mitigate influenza outbreaks in confined military settings [18].

Globally, ARIs beyond influenza have consistently disrupted organizational productivity in military settings, emphasising the universal challenges posed by communal living and intense operational demands. Among troops of the Polish Military Contingent deployed overseas from 2003 to 2005, ARIs were the leading cause of outpatient morbidity, with incidence rates ranging from 45.6-54.8 cases per 100 soldiers, significantly disrupting training schedules and productivity [19]. Similarly, respiratory illnesses affected 69.1% of US military personnel deployed to Iraq and Afghanistan from 2003 to 2004, which often resulted in missed patrols and transient decreases in operational efficiency [20]. Notably, the incidence of ARI doubled during the combat phase, reflecting the heightened susceptibility of military personnel under physically and mentally demanding conditions [20]. Additionally, a systematic review of 46 military studies in 17 countries observed a higher incidence of COVID-19 in military personnel on deployment and those who had overseas exposure, amplifying the risks faced by military personnel where operational stressors and novel environmental exposures exacerbate the burden of ARIs, including COVID-19 [21]. These findings align with our study results, which showed no significant differences in medical absenteeism across pathogens, underscoring the need to adopt prevention strategies targeting the broader spectrum of ARIs rather than focusing primarily on influenza.

A multipronged approach to ARI prevention, integrating vaccination programmes, targeted NPIs and the strategic use of therapeutics, is essential for military personnel. Vaccination has proven highly effective; for instance, the H1N1-2009 influenza vaccination programme in Singapore reduced the weekly cases by 54% among military personnel post vaccination [22]. However, vaccines alone are insufficient, as demonstrated by our study findings. The US military has successfully implemented cohorting strategies in basic training facilities, dividing personnel into smaller, stable groups to limit the exposure and spread of ARI [1]. Complementing these NPIs, personal protective measures such as mandatory mask-wearing during outbreaks or in enclosed, high-density environments, coupled with educational campaigns on respiratory hygiene, could further reduce the ARI transmission [1]. Similarly, Poland’s military experienced peaks in ARI during troop rotations, highlighting the need for stricter isolation protocols and improved acclimatization strategies for personnel entering new environments [19]. Insights from the systematic review on COVID-19 in military settings also emphasised the utility of digital tools, such as contact tracing applications, in effectively managing outbreaks [21]. Therapeutics are another critical component of ARI management. A study in Singapore during the H1N1 influenza outbreak in 2009 demonstrated that early use of oseltamivir significantly reduced the risk of hospitalization and severe outcomes, even in young and healthy military populations [23]. Enhanced vaccination coverage for pathogens with available vaccines (ie, influenza, SARS-CoV-2), rapid isolation of infected personnel, and timely therapeutic interventions collectively contribute to reducing the overall ARI burden. By focusing on overall respiratory health rather than targeting specific pathogens, these strategies will help create a more resilient military workforce.

The findings of our study, while focused on a single SAF military camp, have broader implications for the military and other high-density living settings globally. The consistent alignment of our findings with other military studies, such as those conducted in US, Polish, and Singaporean military contexts, highlights the relevance of our findings across varied geographic and operational contexts. Our study findings are generalizable to other SAF camps or military units with similar profiles, where young men live and work together in close quarters, as these shared conditions contribute significantly to ARI transmission. Additionally, these findings may offer insights for surveillance and ARI prevention measures in other high-density living environments, such as dormitories, where communal living and close interactions similarly increase the risk of ARI outbreaks. Expanding surveillance efforts to multiple camps with different demographics and training intensities, as well as integrating findings with civilian studies, could further enhance the applicability of these findings to both military and nonmilitary populations.

### Strengths

The study used data from comprehensive surveillance with the BioFire FilmArray Respiratory Panel, which enabled the detection of a wide range of respiratory pathogens. The large sample size allowed for accurate determination of pathogen prevalence in the military context. Additionally, the study’s focus on medical absenteeism bridges the gap between clinical outcomes and organizational impact, making the results actionable for health policy and military planning.

### Limitations

First, excluding coinfections from the analysis may have overlooked the complexity of pathogen interactions. Coinfections often exacerbate illness severity or prolong recovery time, and their exclusion may have led to an underestimation of the duration of MA. However, the number of coinfections in our study was relatively small, and their exclusion is unlikely to have significantly impacted the overall results or conclusions. Second, the small number of bacterial ARI cases limited our understanding of their impact on organizational productivity. Bacterial pathogens, such as *Mycoplasma pneumoniae*, are known to cause severe symptoms and potentially longer MA. Without sufficient data, their role in contributing to productivity losses remains unclear. Third, the reliance on self-reported data for fever and medical certificates or light duty duration introduces the potential for recall bias. Lastly, while our study is generalizable to SAF units or camps with similar profiles (ie, young men in close quarters), its findings may not apply to camps with different demographics or training intensities.

Future studies should address these limitations by including coinfection cases to better understand the interplay between different pathogens and their combined impact on ARI severity and duration of medical absenteeism. Expanding surveillance to multiple military camps and similar high-density living settings would enhance generalizability of findings and account for variations in living conditions, vocations, and operational structures. This broader approach could help identify high-risk groups or settings and guide the implementation of targeted interventions. Additionally, longitudinal studies tracking the same cohort over multiple ARI seasons could provide insights into the effectiveness of preventive measures, such as influenza vaccinations, NPIs, and the impact of digital health tools such as the upcoming Data Analytics for Soldier Health (DASH 2.0) system in the SAF. The integration of iDASH2.0 into future electronic medical records could enable real-time monitoring of ARI trends, identification of seasonal trends in pathogen prevalence, and assessing waning immunity post vaccination. Finally, studies focusing on bacterial ARI cases should explore their clinical and operational impact using advanced diagnostic tools and longitudinal study designs, paving the way for antibiotic stewardship programmes.

### Conclusion

This study highlights the significant burden of ARIs in military settings, with influenza contributing to longer medical leave compared to noninfluenza pathogens, yet no differences in the overall duration of medical absenteeism across pathogens. These findings underscore the need for a multipronged approach to ARI prevention that goes beyond vaccination programmes to include targeted nonpharmaceutical interventions and therapeutics. By addressing the identified gaps and building on the strengths of this study, future research should explore the impact of coinfections and bacterial ARIs, seasonal trends, and the long-term effectiveness of preventive measures across multiple military camps in Singapore. This will enhance generalizability and inform robust, evidence-based policies to optimize the health and productivity of servicemen. Ultimately, integrating vaccination programmes, NPIs, and therapeutics into a cohesive ARI management framework will strengthen the SAF’s resilience against respiratory threats and ensure sustained organizational productivity.

## Abbreviations

ARI: acute respiratory infection
FRI: febrile respiratory illness
HEV: human enterovirus
HRV: human rhinovirus
HRV/HEV: human rhinovirus/human enterovirus
IRB: institutional review board
NPI: nonpharmaceutical intervention
OR: odds ratio
SAF: Singapore Armed Forces
